# Value of Radiodensity Determined by Enhanced Computed Tomography for the Differential Diagnosis of Lung Masses

**DOI:** 10.5812/kmp.iranjradiol.17351065.3128

**Published:** 2011-11-25

**Authors:** Min Xie

**Affiliations:** 1 Department of Radiology, Second People’s Hospital of Wuxi, Wuxi, P.R. China

**Keywords:** Tomography, X-Ray Computed, Lung, Tuberculosis, Pulmonary

## Abstract

**Background:**

Lung masses are often difficult to differentiate when their clinical symptoms and shapes or densities on computed tomography (CT) images are similar. However, with different pathological contents, they may appear differently on plain and enhanced CT.

**Objectives:**

To determine the value of enhanced CT for the differential diagnosis of lung masses based on the differences in radiodensity with and without enhancement.

**Patients and Methods:**

Thirty-six patients with lung cancer, 36 with pulmonary tuberculosis (TB) and 10 with inflammatory lung pseudotumors diagnosed by CT and confirmed by pathology in our hospital were selected. The mean ± SD radiodensities of lung masses in the three groups of patients were calculated based on the results of plain and enhanced CT.

**Results:**

There were no significant differences in the radiodensities of the masses detected by plain CT among patients with inflammatory lung pseudotumors, TB and lung cancer (P > 0.05). However, there were significant differences (P < 0.01) between all the groups in terms of radiodensities of masses detected by enhanced CT.

**Conclusions:**

The radiodensities of lung masses detected by enhanced CT could potentially be used to differentiate between lung cancer, pulmonary TB and inflammatory lung pseudotumors.

## 1. Background

Lung masses are one of the most common findings on chest radiographs. However, lung masses are often difficult to differentiate in normal clinical practice. The histology of a mass is important for determining its staging classification, the subsequent selection of appropriate therapies and for evaluating the prognosis. The availability of computed tomography (CT) allows radiologists to better characterize many lung masses. However, in some difficult cases (with similar interfaces, morphology and inner densities), transbronchial lung biopsy, CT-guided percutaneous biopsy or surgery are required to make a diagnosis. Further, non-invasive methods for differentiating lung masses are therefore required. Some lung masses such as inflammatory lung pseudotumors and lung cancer have different structures and blood vessels and may consequently appear differently on plain and enhanced CT. Their radiodensities may also differ between plain and enhanced CT.

## 2. Objectives

This study investigated the differences in radiodensities detected by plain and enhanced CT, as a means of differentiating between lung masses of different etiologies. The study was continued for about 3 years from 2006 to acquire a large enough sample size. Some relevant studies on solitary pulmonary nodules and CT were considered [1][2].

## 3. Patients and Methods

### 3.1. Patients

Thirty-six patients with lung cancer, 36 with pulmonary tuberculosis (TB) and 10 with inflammatory lung pseudotumors, all diagnosed and pathologically confirmed by transbronchial lung biopsy, CT-guided percutaneous biopsy or surgery in our hospital from 2006 to 2009 were selected. Complete clinical data and CT information were available for all patients. The lung cancer group included 22 males and 14 females (age range, 34-77 years), the lung TB group included 19 males and 17 females (age range, 26-58 years) and the lung pseudotumor group included four males and six females (age range, 19-55 years). All patients were selected according to the following criteria: [1] Each patient had a round or oval-shaped mass in the lungs with a diameter larger than 1 cm, with no evidence of metastasis or atelectasis in the lungs. Patients with TB lesions resembling pneumonia or with satellite lesions around the lung masses were excluded from the study. Patients with evidence of calcification of the lung mass were also excluded [2]. Absence of contraindications to the administration of contrast material [3]. Ability to cooperate with the procedure [4]. Complete clinical and pathological information available. Patients with serious basic lung diseases, significant pleural effusions, heart failure or hepatic insufficiency were excluded from the study.

### 3.2. CT Examination

A Hispeed CTi single slice spiral CT scanner (GE, USA) was used. All patients provided their informed consent in accordance with the 1975 Declaration of Helsinki. The protocol complied with the Declarations of Helsinki and Tokyo and was approved by our institutional Ethics Committee. All patients received plain and enhanced examination with a scanning thickness of 5-10 mm. Contrast medium (Ultravist) was injected at 1.5-2.0 mL/kg at a velocity of 3.0 mL/s. Scanning began about 60-70 s after injection of the contrast medium. The scanning parameters were consistent throughout the study. The sizes of the masses were measured on the CT scanning images. The radiodensities of the masses were measured after scanning by first identifying the most enhanced area, then selecting several circles within that area, each representing about 5-10% of the total area. The mean radiodensity value was based on the densities from all the circles excluding necrotic areas, image artifacts, calcification or cavities. Measurements were made by two experienced chief radiologists who were blinded to the pathology results. The radiodensities of masses detected by plain and enhanced CT in each patient were recorded. The mean ± SD from each plain scan, the mean ± SD from each enhanced scan and the mean ± SD enhancement (value detected by enhanced CT minus the value detected by plain CT) were calculated for the patients in each group. The data were analyzed using F tests and SNK-q tests and the radiodensities of masses detected by plain and the enhanced CT were compared among the groups. P < 0.05 were considered to indicate a significant difference.

## 4. Results

**Figure 1:**
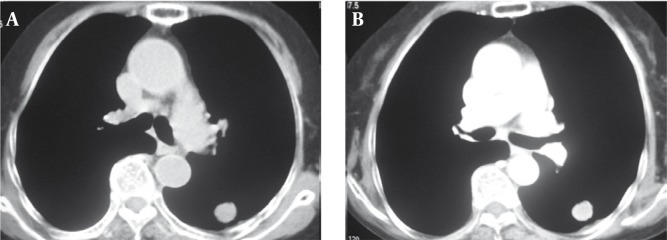
Computed Tomography of a 66-Year-Old Man Presenting With Chest Pain and Cough. A, Plain CT scan shows a round mass in the left lung. B, Enhanced CT scan shows slight homogeneous enhancement in the mass which was verified to be lung cancer.

Comparing enhanced CT with plain CT scans, 19 of the 36 lung cancer cases showed moderate and inhomogeneous enhancement, while the other lung cancer cases showed slight enhancement (Figure 1). Twenty-five of the 36 pulmonary TB cases showed slight enhancement and 11 cases showed moderate, homogeneous or inhomogeneous enhancement. Four of the 10 cases of inflammatory lung pseudotumors showed obvious and homogeneous enhancement, five showed moderate and homogeneous or inhomogeneous enhancement and one showed slight enhancement. There was no significant difference in the radiodensities of masses detected by plain scans between any of the groups (P > 0.05). However, the radiodensities of masses detected by enhanced CT differed significantly among all three groups (P < 0.01). These data are shown in Table 1, 2, 3, 4 and 5

**Table 1:** Radiodensities of Masses in Patients With Lung Cancer, Pulmonary TB and Inflammatory Lung Pseudotumors Measured by Plain CT a

**Radiodensity (HU)**	**Lung Cancer**	**Pulmonary TB b**	**Inflammatory Lung Pseudotumor**
Minimum	19.5	29.8	38.5
Maximum	52.7	59.2	49.6
Mean ± SD	39.3 ± 3.0	43.2 ± 10.2	42.4 ± 3.0

^a^ Although the mean values were similar in the three groups, the SD was considerably higher in the TB group compared with the other two groups.

^b^ Abbreviation: TB, Tuberculosis

**Table 2:** Radiodensities of Masses in Patients With Lung Cancer, Pulmonary TB and Inflammatory Lung Pseudotumors Measured by Enhanced CT a

**Radiodensity (HU)**	**Lung Cancer **	**Pulmonary TB b**	**Inflammatory Lung Pseudotumor**
Minimum	46.5	38.2	75.4
Maximum	79.5	67.5	97.5
Mean ± SD	70.3 ± 8.7	52.0 ± 6.7	85.5 ± 6.3
Enhancement, Mean ± SD	28.7 ± 11.2	8.1 ± 5.5	42.1 ± 8.5

^a^ The mean radiodensities, enhancement (mean ± SD) differed significantly among the groups. The mean enhancement was lowest in the pulmonary TB cases.

^b^ Abbreviation: TB, Tuberculosis

**Table 3:** Comparison of Radiodensities Among Lung Cancer, Pulmonary TB and Inflammatory Lung Pseudotumor Cases Using Plain CT a

	**MS b**	**F value**	**P value**
Between groups	142.7	2.836	> 0.05
In groups	50.3	-	-

^a^ There was no significant difference by F tests in radiodensities determined by plain CT between any of the groups.

^b^ Abbreviation: MS, Mean Square

**Table 4:** Comparison of Radiodensities Among Lung Cancer, Pulmonary TB and Inflammatory Lung Pseudotumor Cases, Using Enhanced CT a

	**Pulmonary TB**	**Inflammatory Lung Pseudotumor**	**Lung Cancer**	**P value**
Lung cancer	q = 9.2	-	-	< 0.01
Pulmonary TB b	-	q = 15.6	-	< 0.01
Inflammatory lung pseudotumor	-	-	q = 7.1	< 0.01

^a^ Significant differences in radiodensities determined by enhanced CT were identified between all groups by SNK-q test analysis.

^b^ Abbreviation: TB, Tuberculosis

**Table 5:** Comparison of Enhancement Radiodensities Among Lung Cancer, Pulmonary TB and Inflammatory Lung Pseudotumor Cases Using Enhanced CT a

	**Pulmonary TB**	**Inflammatory Lung Pseudotumor**	**Lung Cancer**	**P value**
Lung cancer	q = 35.8	-	-	< 0.01
Pulmonary TB b	-	q = 17.5	-	< 0.01
Inflammatory lung pseudotumor	-	-	q = 6.0	< 0.01

^a^ Significant differences in enhancement radiodensities determined by enhanced CT were identified between all groups by SNK-q test analysis.

^b^ Abbreviation: TB, Tuberculosis

## 5. Discussion

Lung masses are common findings in chest imaging. They may be benign or malignant; pulmonary TB, lung hamartomas and inflammatory lung pseudotumors are benign, while squamous cell carcinomas, adenocarcinomas and small and large cell carcinomas are malignant. Benign masses often appear spherical with smooth margins, while malignant lung cancers often appear lobular. Some masses have specific manifestations on CT images, such as cavities or satellite lesions. TB may manifest with cavities surrounded by satellite lesions and fiber bundle shadows; lung hamartomas have fatty elements and popcorn calcifications; malignant masses may include eccentric cavities and have indistinct margins with sunken pleura accompanied by enlarged lymph nodes at the hilus and mediastinum. Central lung cancer may be associated with an abnormal bronchus. Patients with pulmonary TB may suffer from low fever, night sweating and hemoptysis, while patients with lung cancer also suffer from symptoms such as hemoptysis, irritable cough and chest pain. All these clinical manifestations are helpful for the differential diagnosis of lung masses.

However, masses may be difficult to differentiate when the symptoms and the shapes or densities of the masses on CT images are similar with no distinctive cavities or satellite lesions. Nevertheless, the different pathologic structures and blood vessel contents may result in differences in detectability by enhanced CT. The results of the current study showed no significant differences in radiodensities of masses detected by plain CT between any of the groups. This could be because of the strict patient inclusion criteria and the exclusion of patients with calcification or else in the lung masses. The variation in detected radiodensities was larger in the TB group than in the other groups, because of the large variations in necrotic tissue in TB. Previous studies have suggested that an enhancement of less than 20 Hounsfield units (HU) is likely to represent a benign mass, while 20-60 HU suggests malignancy and an enhancement higher than 60 HU may indicate an inflammatory tumor [1][2][3][4]. In this study, the mean enhancement in cases with pulmonary TB (benign masses) was 8.1 HU (< 20 HU), in lung cancer cases (malignant masses) the mean enhancement was 28.7 HU (20-60 HU), and in cases of inflammatory lung pseudotumors (inflammatory tumors) this figure was 42.1 HU (< 60 HU). The lower than expected value for inflammatory lung pseudotumors may have been due to the presence of multiple cells and the pathologic phase.

Angiogenesis varies greatly between benign and malignant masses [5][6][7][8][9][10][11]. Based on pathologic analysis, pulmonary TB includes tissues with structural necrosis and few blood vessels and internal enhancement is subsequently not obvious. However, the blood supply to the membrane is better and complete or incomplete membrane enhancement is thus a morphologic feature of TB. Lung cancer shows pathologic proliferation and inappropriate angiogenesis with the establishment of vascular networks that support tumor growth. Tissues inside lung cancer masses grow inhomogeneously, some having rich blood supply and others poorer blood supply. Inflammatory pseudotumors are actually granulomas formed by inflammatory proliferation. They include many capillaries and a rich blood supply, hence explaining the high degree of enhancement on CT. However, during the progression from an active to a chronic pseudotumor, the fibrous content increases and the vascular content decreases. Theoretically, the degree of enhancement of the mass depends on the quantity of contrast medium entering the extra vessel space and the number of vessels within the mass [3] accounting for the wide variation.

Four types of enhancement may generally be recognized:

1) homogeneous

2) inhomogeneous

3) central

4) marginal

The enhancement in lung cancer cases in this study was mainly moderate and inhomogeneous, related to cancer necrosis and the inhomogeneous blood supply. Enhancement in pulmonary TB was mainly slight or moderate, as a result of internal necrosis and the inhomogeneous supply of blood vessels. Enhancement in inflammatory lung pseudotumors was mainly obvious and inhomogeneous, because the inflammation results in a mass with a relatively homogeneous blood supply. The results of this study suggest that lung masses of similar shapes and densities may be differentiated on the basis of enhanced CT, allowing improved preoperative diagnostic accuracy, accordingly aiding the selection of appropriate surgical treatment.
